# Successful Removal of a Large Ovarian Tumor

**Published:** 1886-04

**Authors:** Henry D. Ingraham

**Affiliations:** Prof. of Diseases of Women and Children, Medical Department, Niagara University


### Clinical Reports.

### Successful Removal of a Large Ovarian Tumor.—Recovery.

By Henry D. Ingraham, M. D.,

Prof. of Diseases of Women and Children, Medical Department, Niagara University.

Ovariotomy is so frequently performed, and usually with success by the surgeon of average ability, who is careful in regard to all the details of the operation, that I would not think of reporting this case were it not for two or three points of unusual interest connected with it.

On the 11th of last June, Miss T—— B—— was admitted to the gynæcological department of the Buffalo Hospital of the Sisters of Charity. She was about medium height, slight in figure, with an enormously distended abdomen. Although she was only twenty-two years old, she looked twice that number of years, due to the peculiar expression of countenance, the *facies ovarina*, which was particularly well marked in her case. The patient had always been in good health until about a year and a half previously.

Her parents were both living and in good health. She first menstruated at fourteen and had been regular ever since, except during the past fifteen months, the flow being normal in every respect. Her appetite and digestion had been good, and bowels regular, until within the last few months. Her father is a farmer, and she had worked on his farm a greater portion of the time during the summer since she was sixteen years old, until the beginning of her sickness. As would be quite natural, she attributed her illness to the out-door work, although she remarked that she preferred work on the farm rather than in the house, and wished she were a man — a very natural wish, considering her condition. About eighteen months previous, she noticed an increase in the size of the abdomen, and began to suffer from dragging sensations in the pelvis, and pain in the back extending down the thighs, together with irritation of the bladder, a frequent desire to urinate, accompauied with vesical tenesmus, constipation and dysmenorrhœa. These symptoms continued to increase, and soon hemorrhoids were developed, and, after three months, menstruation stopped.

A month or so later, she consulted the family physician, Dr. Trull, of Williamsville, who, after a careful examination, told her that she had an ovarian tumor. The abdomen continued to enlarge, the pain in the back became more severe, the vesical irritation increased, and most of the symptoms usual to an ovarian tumor were quite pronounced. About thirteen months after the first appearance of the tumor she was tapped, and three quarts or more of fluid drawn, which reduced the size of the abdomen considerably. Patient menstruated a few days later, but the flow was scanty and painful. She felt more comfortable for a short time, but soon the disagreeable symptoms began to return; and, feeling that she could not last long as she was, upon the advice of her physician, she came to the hospital to have the tumor removed.

Upon examination, the abdomen was found to be greatly distended up to the ensiform cartilage, and symmetrical. The chest was conical; the superficial veins were enlarged and prominent; the *lineæ albicantes* well marked; the umbilicus protruding; the surface of the abdomen smooth, tense and shining; the follicles around the nipple were unequally developed, and of the same color as that of the areola. Only a portion of the upper part of the tumor was elastic and fluctuating, being more firm and dense in the middle and lower portions. Above, and to the left of the umbilicus, was a firm rounded body which felt like the head of a full-grown fœtus. Below, and continuous with this, the growth was hard, firm, dense, irregular and immovable at the lower portion, which was decidedly nodulated. The abdomen being filled with the growth, changing the position of the patient, made no change in the position of the tumor. There was dullness on percussion over the whole abdomen, except a portion of the right iliac region, which was partially resonant. Neither the aortic impulse, nor the sound which usually accompanies it, could be heard. Vaginal examination revealed a tumor in front of the uterus, that organ being crowded over to the right side and firmly fixed in the pelvis. The cervix was normal, except a trifle shorter than natural; the uterus was slightly enlarged, measuring almost three inches in depth. Strong pressure upon the tumor raised it a little, and also raised the uterus a trifle. There was no downward movement of the tumor during inspiration, or upward movement during expiration, as is often the case where adhesions do not exist.

The patient was carefully examined by Prof. Cronyn, Dr. Banta and myself, and, although there were several symptoms which would indicate that the growth was not ovarian, yet we were obliged to rule out everything else and conclude that it was an ovarian polycyst. I therefore decided to remove the growth. The patient was put in as good condition as possible during the next few days. The operating room, and also the room in which she was to remain afterwards, were thoroughly cleaned and then sprayed for twenty-four hours previous to the operation with a 1 to 2000 solution of corrosive sublimate. On the 20th day of June, at 12 M., I proceeded with the operation, and was assisted by Prof. Daniels, Drs. Banta and F. H. Potter, the latter giving the ether. Profs. Cronyn, Davidson, Stockton, Heath, Fell, Drs. Trull, Crego, Campbell, Frederick, Clark, Jewett, Green, Berkes, Messrs. Murphy and Hill, hospital internes, and three or four medical students, were also present during the operation.

The patient was brought under the ether very quickly, when the abdomen was cleaned by being sponged with a mixture of turpentine and alcohol, and the hair was shaved off. An incision was made along the *lineæ alba* from the umbilicus to a point a little above the symphysis pubis, and the tumor exposed. A trocar was introduced into its upper portion, and about four quarts of a thin light brown liquid drawn off, when the flow ceased.

Upon removing the trocar and opening the sack sufficiently to admit the hand, it was found that the contents of the largest cyst had been evacuated. Several smaller cysts were broken into and evacuated, some of them with great difficulty, as the walls were thick and strong. The tumor was composed of a large number of cysts, but with the exception of the one first opened, none of them contained much fluid, some of them not more than an ounce or two, others five or six ounces. There were any number of small cysts, the size of a robin's egg, or even smaller, with firm, dense walls that could not be broken with the finger, and, when cut open, they were found to contain a thick, creamy-looking substance. In some cases the contents looked very much like thick, cooked starch. The fluid in some of the larger cysts was nearly the same in appearance. Firm, strong adhesions were found at the left and upper portion of the tumor, requiring unusual force to sever them. In fact, I was afraid that I should not be able to break them without using too much force for the good of the patient, but succeeded, however, without doing any injury to the surrounding parts.

Owing to the small amount of fluid in the tumor, it was found impossible to remove it through the opening already made, and the incision was extended upward on the left of the umbilicus nearly to the ensiform cartilage.

The upper portion of the tumor was raised out of the abdomen, and the pelvic adhesions, being less firm than the others, were quite easily broken. The pedicle was short, broad and thick, as is usual in polycysts. It was secured by passing a double carbolized silk ligature through it, and tying either half. The tumor was then severed from it, half an inch outside of the ligature, and the stump seared with the thermo-cautery. The other ovary was examined and found to be healthy, when the ends of the ligature around the pedicle were cut off short, and the abdomen was thoroughly cleaned by carefully sponging with warm carbolized water.

Upon removing the tumor, some of the fluid from one of smaller cysts escaped into the abdominal cavity, but doing no harm to the patient, as the result demonstrated. The greatest care and attention was given to the "toilet of the abdomen," believing it to be of the utmost importance.

The external wound was closed by six silver sutures, the stitches being passed from within outwards; that is, the needle was passed through the peritoneum first; several superficial silk stitches were taken between the wire ones; strips of adhesive plaster, about six inches long, were placed across the abdomen, and a dressing of antiseptic jute and gauze was used, it being retained in place by long strips of adhesive plaster encircling the abdomen.

About 2 P. M. the patient was put into bed and covered with warm blankets, and bottles of hot water were put to her feet.

The remainder of the history of the case will be taken from the record kept by the resident, Mr. Murphy.

June 20th, 8 P. M.—Patient rallied without any unpleasant symptoms; slight vomiting from ether; small pieces of ice given every half hour; mouthfuls of hot water every hour; morphine, ¼ grain, hypodermically; pulse, 122; temperature, 99; urine drawn.

21st, 8 A. M.—Patient slept well last night; no pain or vomiting; pulse, 124; temperature, 99; morphine, ¼ gr., hypodermically; urine drawn; beef tea given in small quantity. 12 M.—Patient comfortable; beef tea in small quantities every two hours; pulse, 120; temperature, 99½; morphine, ¼ gr., hypodermically; urine drawn. 8 P. M.—Patient quiet; pulse, 110; temperature, 100½; morphine, ¼ gr., hypodermically; urine drawn; 12 midnight.—Patient sleeping.

22d, 8 A. M.—Comfortable; pulse, 106; temperature, 99; morphine, ¼ gr., hypodermically.

Thus the record runs, except less morphine was given, and on the 23d, a one grain pill of opium was substituted for the hypodermic use of the morphine, and this was continued two or three times a day, as necessary. At no time were there any unpleasant symptoms whatever.

June 27th, 12 M.—The record reads: Dressings removed under spray; wound healed by first intention; stitches removed, except the superficial silk ones.

This was the first time the dressing had been changed, and the result was very satisfactory.

Antiseptic gauze was placed on the wound, and long strips of adhesive plaster applied, encircling the body. The bowels had been moved by enema the day before. Small quantities of milk and rice, corn starch, etc., were given now. Previous to this time, no nourishment but beef tea had been allowed. One grain of opium was given at bedtime for the following two or three nights, and, on July 1st, the superficial stitches were removed and the abdomen encircled by a flannel bandage.

A week later she was allowed to sit up a portion of the time, and in a few days rode home, a distance of fourteen miles.

The weight of the tumor, both the solid and liquid portions, was sixty-four pounds.

The points to which I wish to call particular attention are these:

First.—The surface of the abdomen was smooth, tense and shining, which is not usual in ovarian tumors.

Second.—The uterus moved slightly with the tumor, which it is said not to do, unless the growth be a fibroid of the uterus. In this case, it was doubtless due to the removal of the pressure from the uterus, and, perhaps, to the adhesions which were found to exist in the pelvis, although not to the uterus itself.

Third.—To the fact that although some of the fluid escaped from the tumor into the abdominal cavity, yet the patient made as good a recovery as was possible had not this unavoidable accident occurred. The idea that the escape of the fluid into the abdomen necessarily does harm, or greatly lessens the chance of the patients recovery, is not as generally believed as formerly, if the abdomen be thoroughly and properly cleaned. In this case it was very carefully cleaned by sponging with warm carbolized water.

Fourth.—To the fact that for twelve hours after the operation nothing was given the patient, except one or two tablespoonfuls of hot water every hour, and small pieces of ice every half hour or so. I find that when patients are treated in this manner after taking ether, they have much less nausea and vomiting, and are much better the following day than when they are given nourishment.

Fifth.—Also, that only beef tea, in small quantities, was given the six days following the operation, when an enema was administered and the bowels moved; then nourishment was given more freely. It is my experience that, in all gynæcological operations, the patient makes a much better recovery on a limited and light diet for the first few days. No small amount of credit in the recovery of this patient is due to the house physician, Mr. Murphy, the Sister in charge, and the nurse.

